# Isolating Fe-O_2_ Intermediates in Dioxygen Activation by Iron Porphyrin Complexes

**DOI:** 10.3390/molecules27154690

**Published:** 2022-07-22

**Authors:** Xiaoyan Lu, Shuang Wang, Jian-Hua Qin

**Affiliations:** College of Chemistry and Chemical Engineering, Luoyang Normal University, Luoyang 471934, China; dyyxqy1990@163.com (S.W.); jh_q128105@126.com (J.-H.Q.)

**Keywords:** oxygen activation, iron porphyrin complexes, Fe-O_2_ intermediates, enzymes, superoxo

## Abstract

Dioxygen (O_2_) is an environmentally benign and abundant oxidant whose utilization is of great interest in the design of bioinspired synthetic catalytic oxidation systems to reduce energy consumption. However, it is unfortunate that utilization of O_2_ is a significant challenge because of the thermodynamic stability of O_2_ in its triplet ground state. Nevertheless, nature is able to overcome the spin state barrier using enzymes, which contain transition metals with unpaired *d*-electrons facilitating the activation of O_2_ by metal coordination. This inspires bioinorganic chemists to synthesize biomimetic small-molecule iron porphyrin complexes to carry out the O_2_ activation, wherein Fe-O_2_ species have been implicated as the key reactive intermediates. In recent years, a number of Fe-O_2_ intermediates have been synthesized by activating O_2_ at iron centers supported on porphyrin ligands. In this review, we focus on a few examples of these advances with emphasis in each case on the particular design of iron porphyrin complexes and particular reaction environments to stabilize and isolate metal-O_2_ intermediates in dioxygen activation, which will provide clues to elucidate structures of reactive intermediates and mechanistic insights in biological processes.

## 1. Introduction

Dioxygen (O_2_) is a primary component of the atmosphere and is essential in life processes. The biochemical processes that mediate the highly exergonic four-electron reduction of oxygen to water with an enthalpy change of −80 kcal/mol provide the energy for sustaining aerobic organisms [1]. With a redox potential of 0.815 V vs. NHE in water at pH 7 and 25 °C, the overall four-electron reduction of O_2_ is thermodynamically favorable [2]. However, the activation of O_2_ is kinetically hindered because of its triplet ground state, which makes direct combination of O_2_ with typical organic compounds spin-forbidden [3,4,5]. To overcome this difficulty, nature has evolved a wide variety of enzymes, which contain transition metals (e.g., Fe, Mn, Cu, and Co) with unpaired *d*-electrons facilitating the activation of O_2_ by metal coordination, to carry out various vital biological processes [6]. The function of transition metals is not only to serve as O_2_ binding sites for transport, but also to serve as active centers where they can carry out multi-electron redox process that ultimately result in the incorporation of oxygen in organic substrates [7].

**Figure 1:**
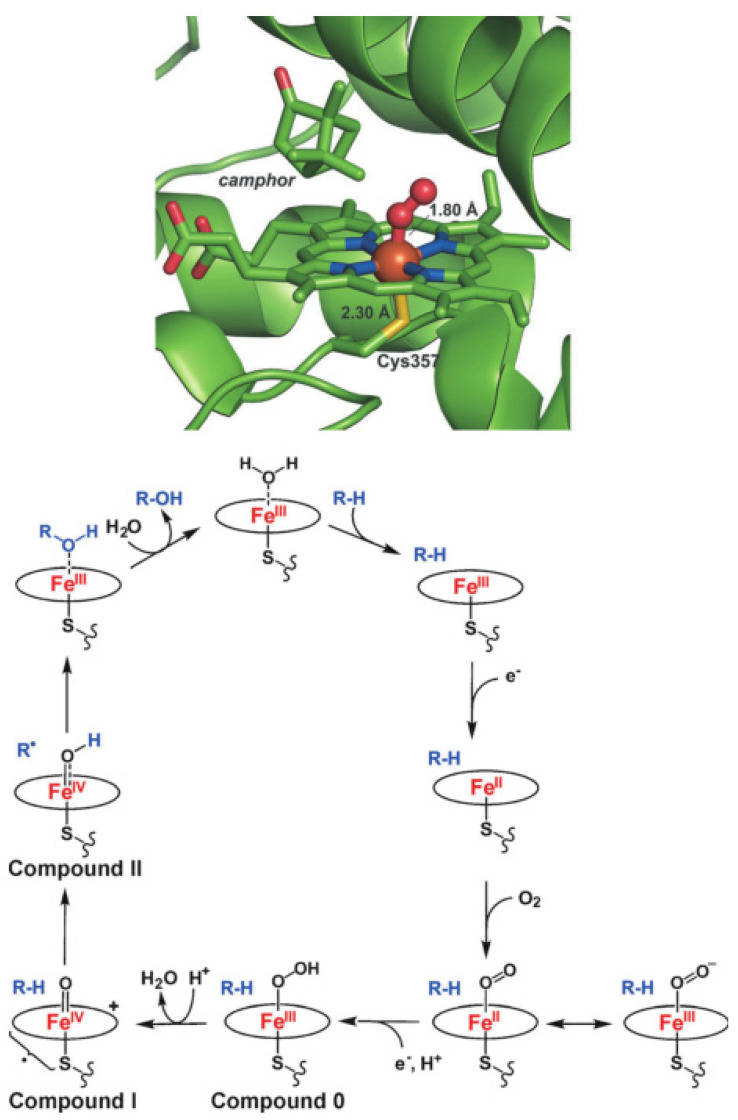
O_2_ bound to the ferrous heme in the active site of cytochrome P450 (**top**); the catalytic cycle of cytochrome P450 (**bottom**). Reprinted with permission from Ref. [9]. Copyright 2014, WILEY-VCH Verlag GmbH.

The binding of O_2_ to ferrous porphyrins constitutes the first step in the biological function of a majority of heme enzymes. Processes involving cleavage of the O-O bond require proteins with specific structural and chemical features designed to facilitate O_2_ activation [7]. Cytochrome P450, one of the important dioxygen-activating heme enzymes, has thrown light on the catalytic cycles of O_2_ activation and oxygen transfer reactions [8,9,10,11,12]. The consensus mechanism for O_2_ activation by cytochrome P450 is shown in Figure 1 [9]. The spin-state of the six-coordinate ferric complex at the active site in cytochrome P450 is low-spin, which turns into a high-spin five-coordinate ferric complex upon binding of the substrate to remove the aqua ligand. One-electron reduction of this high-spin five-coordinate ferric species generates a high-spin ferrous compound, which is quite activated for O_2_ binding to give an iron (III)-superoxo intermediate. Then, a second one-electron reduction occurs to form an iron (III)-peroxo complex, which turns into an iron (III)-hydroperoxo complex (Compound 0) upon protonation. Heterolytic cleavage of the O-O bond of Compound 0 produces an iron (IV)-oxo porphyrin *π*-cation radical intermediate (Compound I) and one molecule of water [10]. The generally accepted mechanism for C-H bond activation of alkanes by Compound I is the “oxygen-rebound” mechanism [11], where the H-atom of the substrate is abstracted by Compound I to generate an iron (IV)-hydroxo species and a carbon radical that recombine to form alcohol and the ferric heme resting state.

Metal-dioxygen species are crucial intermediates in the O_2_ activation cycle of metalloenzymes, and their redox reactivities are modulated by the nature of metal ions and ligands in metalloenzymes [13,14,15,16,17,18,19,20,21,22,23,24,25,26]. In living organisms, the metalloporphyrin sites are surrounded by the protein superstructure, which is conducive to avoiding the involvement of metalloporphyrin active sites in unwanted side reactions [13,15,27,28,29,30]. However, the complexity of the surrounding protein superstructure restricts access to the metalloporphyrin active sites, resulting in the big challenge in studying the intermediates in O_2_ activation by metalloenzymes. To deal with this big challenge, the last several decades have seen numerous researchers turn towards the development of molecular model complexes with heme and nonheme ligands that mimic the local coordination environment of the active site in enzymes [31,32,33,34,35,36,37]. Due to the simplicity of molecular model complexes, the metal–dioxygen intermediates can be stabilized and directly characterized using current techniques.

The heme cofactor, being made of an iron-bound porphyrin, is at the active site of many metalloproteins and carries out oxygen transport, storage, electron transfer, and oxygenation reactions [38,39,40]. Thus, due to the bioavailability of iron porphyrins in nature, iron porphyrins have been well-studied. The successfully isolated and characterized metal-dioxygen intermediates in iron porphyrin complexes can make us understand physicochemical properties of the metalloenzymes and study the correlation of the structural properties of the active sites and their reactivities in well-controlled environments. In this review, we focus on a few examples of these advances with emphasis in each case on the particular reaction conditions and design of iron porphyrin complexes to stabilize and isolate Fe-O_2_ intermediates in O_2_ activation, which will provide clues to elucidate structures of reactive intermediates and mechanistic insights in biological processes for the design of effective catalysts.

## 2. Nature of Iron–Dioxygen Bond

**Figure 2:**
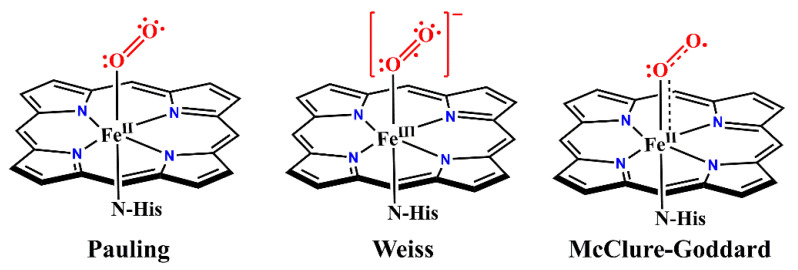
Three models of the iron–dioxygen bond. Pauling model: Low-spin (*S* = 0) Fe^II^ with singlet O_2_; Weiss model: Low-spin (*S* = 1/2) Fe^III^ antiferromagnetically coupled to an O_2_^•−^; McClure-Goddard model (ozone model): Intermediate-spin (*S* = 1) Fe^II^ antiferromagnetically coupled to triplet O_2_. Reprinted with permission from Ref. [45]. Copyright 2013 American Chemical Society.

The structural and electronic properties of iron–dioxygen adduct are crucial for understanding the O_2_ activation in heme proteins and metalloporphyrins. However, a controversy exists over the nature of Fe-O_2_ bonds in hemoproteins and metalloporphyrins. As early as 1936, Pauling and Coryell reported that oxygenated hemoglobin (oxy-Hb) was diamagnetic and proposed that dioxygen was bound to iron in a bent, end-on mode [41,42]. This geometric feature was confirmed by an oxy−ferrous model compound prepared by Collman and co-workers and by crystal structures of oxy-Hb reported by Shaanan, respectively [43,44]. These pioneering works suggest that the electronic structure of oxy-Hb is described as a Fe^II^-O_2_ adduct, where complete electron transfer from O_2_ to iron has not taken place (Figure 2) [45].

In contrast, Weiss and co-workers propose that the Fe−O_2_ species would be best described as a ferric superoxide complex, where the observed diamagnetism of oxy-Hb is explained by the antiferromagnetic coupling between a doublet superoxide anion O_2_^•−^ (*S* = 1/2) and a low-spin Fe^III^ (*S* = 1/2) to result in an overall spin of *S* = 0 (Figure 2) [45]. Mössbauer studies have lent support to the Weiss model. The Mössbauer parameters of oxy-Hb with isomer shift *δ* = 0.2−0.3 mm/s and quadrupole splitting ΔE_Q_ = 2.00–2.20 mm/s, first measured by Lang and co-workers, are in good agreement with a low-spin ferric center [46,47]. Sharrock and co-workers have given close parameters for oxygenated reduced P450cam, which are *δ* = 0.3159 mm/s and ΔE_Q_ = 2.15 mm/s [48]. Collman and co-workers present infrared data on Fe-O_2_ adducts in “picket-fence” porphyrin (*α*,*α*,*α*,*α*-tetrapivalamidophenylporphyrin, *α*,*α*,*α*,*α*-TpivPP) complexes with 1-methylimidazole (1-MeIm) and 1-tritylimidazole (1-tritylIm) as axial bases. The O_2_ vibration of Fe-O_2_ adducts are achieved using difference techniques between ^16^O_2_, ^18^O_2_, ^16^O-^18^O, and NO with a Fourier transform infrared spectrometer. They compare the obtained *v*_O2_ with known M-O_2_ (M = Fe, Co, Cr, and Ti) complexes and concluded that Fe-O_2_ adducts are best described as Fe^III^-O_2_^•−^ [49]. The Fe^III^-O_2_^•−^ model obtained further support by resonance Raman spectroscopic studies, where a ferric center was indicated by the oxidation state marker band of oxy-Hb (*ν* = 1377 cm^−1^) [50,51,52].

The third model (“Ozone model”), initially suggested by McClure and refined by Goddard, presents the oxy-form electronic structure as an antiferromagnetically coupled intermediate spin ferrous center (*S* = 1) and a triplet dioxygen ligand (*S* = 1) [53,54,55,56]. In a recent report, Haumann and co-workers apply the X-ray spectroscopic techniques and computations on O_2_-bound hemes in myoglobin (Mb) and hemoglobin (Hb) and in porphyrin complexes at 20–260 K [57]. Based on the results, they describe an essentially ferrous, intermediate-spin iron in an ozone-like configuration in O_2_-bound heme compounds, supporting the McClure–Goddard model.

Using generalized valence bond (GVB) wave function, Shaik and co-workers have presented Fe-O_2_ bonding that includes a σ (Fe−O) bond and a *π* (Fe-O_2_) bond between *d*_yz_ (Fe) and *π*_⊥_* (O_2_) with quite small delocalization tails [58]. Thus, they propose one electron transferred from Fe^II^ center to O_2_ to form Fe^III^ center and superoxo O_2_^•−^, that is, the Weiss bonding model. Furthermore, H-bonding capability and the polarity of the protein is able to modulate the O_2_ affinity to consolidate the character of Fe^III^-O_2_^•−^. Although Fe−O_2_ follows the Fe^III^-O_2_^•−^ bonding model from the GVB wave function, the pure VB analysis showed a mixing of the other two models with the Pauling model being the least important, and axial ligands and protein host determined the nature of the Fe−O_2_ bonding. In addition, an iron L-edge X-ray absorption spectroscopy study of a ferric-superoxo “picket fence” porphyrin with 1-methylimidazole (1-MeIm) axial ligand, [Fe(O_2_)(1-MeIm)(*α*,*α*,*α*,*α*-TpivPP)], also strongly supports the key impact of local environment on the nature of Fe−O_2_ bonding [45]. Interestingly, in a recent work of Sarangi and co-workers, the solution-state structure of oxy-Hb is best described as Fe^III^-O_2_^•−^ (Weiss model); however, the crystalline oxy-Hb favors (Fe^II^−O_2_) (Pauling model) through theoretical and spectroscopic studies. This surprising change of electronic configuration with environment is in favor of the multiconfigurational description [59]. Even though the current findings of oxy-heme intermediates are unable to give a simple explanation for their electronic structures, notable recent work has presented the necessity of electron transfer from the iron center to the dioxygen moiety so as to produce oxy-heme-type intermediates, emphasizing the unambiguous validity of the Weiss model [60,61,62,63,64].

## 3. Strategies to Stabilize Iron–Dioxygen Intermediates in O_2_ Activation

### 3.1. Sterically Hindered Metal Porphyrin Complexes

**Scheme 1:**
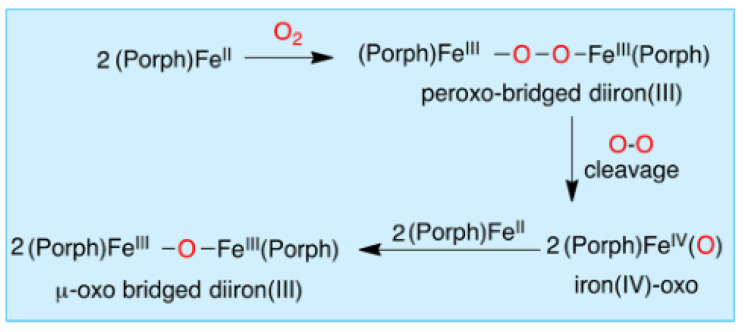
Dioxygen-mediated autoxidation mechanism for ferrous porphyrin complexes. Reprinted with permission from Ref. [68]. Copyright 2016 American Chemical Society.

Biomimetic synthetic iron porphyrin complexes, which have the same core structure as the heme cofactor, are perfect models to study the O_2_ activation. However, a major hurdle presents in simple iron (II) porphyrin complexes, which are facile to undergo bimolecular condensation to form μ-oxo-bridged diiron (III) complexes upon reacting with O_2_ [13]. The single-atom bridged binuclear complexes have been considered as catalytically inert for a long time. However, Sorokin’s group synthesized and spectroscopically characterized a variety of μ-oxo-bridged and μ-nitrido-bridged complexes, which provided superior catalytic properties [65,66,67]. For example, employing μ-nitrido-bridged diiron *meso*-tetraphenylporphyrin [(TPP)Fe^III^(μ-N)Fe^IV^(TPP)], they prepared a highly reactive intermediate, Fe(IV)(μ-N)Fe(IV)(=O) tetraphenylporphyrin cation radical species, which exhibited a very high activity for C–H activation towards alkanes, even including the strongest C–H bond of methane [65]. Until the 1980s, the mechanism for the bimolecular condensation was fully described as shown in Scheme 1 [68], in which the autoxidation proceeds via a peroxo-bridged diiron(III) and the Fe^IV^(O) intermediates [68,69,70,71,72]. Encouragingly, this hurdle has been partially overcome through introduction of sterically hindered substituents onto porphyrin scaffolds, which prevent bimolecular condensation reactions and enable isolation and thorough characterization of heme-O_2_ adducts [73,74,75,76,77,78,79,80]. 

**Figure 3:**
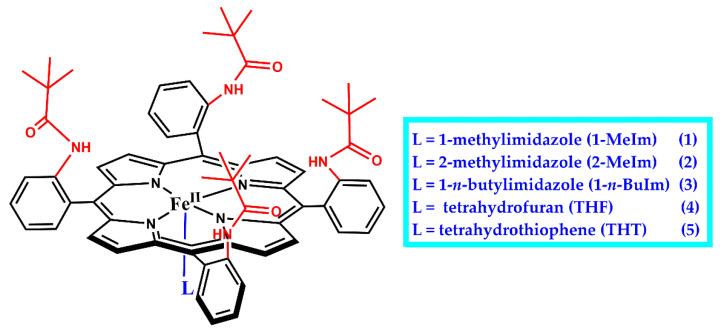
Chemical structures of iron(II) complexes of “picket-fence” porphyrin with substituted axial ligands, [Fe^II^(L)(*α*,*α*,*α*,*α*-TpivPP)].

Much progress in stabilizing an Fe–O_2_ moiety as a functional mimic of an O_2_-bound heme system has been made with sterically protected porphyrin scaffolds such as “picket-fence” porphyrins, which is first implemented by Collman and co-workers to prevent the irreversible bimolecular condensation of iron(II) porphyrin complexes to form μ-oxo-bridged diiron(III) species upon reacting with O_2_. In 1973, Collman and co-workers construct a “picket fence” porphyrin, whose steric bulk on one side creates a nonprotic cavity for the coordination of O_2_ while also protecting O_2_ from bimolecular reactions [60]. *meso*-Tetra(*o*-aminopheny1)porphyrin (H_2_TamPP) is synthesized by SnCl_2_ reduction of *meso*-tetra(*o*-nitropheny1)porphyrin and separated into its four atropisomers. Then, the slowest moving isomer is isolated in 12% yield and its configuration frozen by formation of the amide, *meso*-tetra(*α*,*α*,*α*,*α*-*o*-pivala midephenyl)porphyrin (*α*,*α*,*α*,*α*-H_2_TpivPP) [77]. Treatment with FeBr_2_ affords purple crystals of [Fe^III^Br(*α*,*α*,*α*,*α,*-TpivPP)], which is then reduced to give [Fe^II^(*α*,*α*,*α*,*α*-TpivPP)]. [Fe^II^(*α*,*α*,*α*,*α*-TpivPP)] reacted with 1-methylimidazole (1-MeIm) giving crystalline, diamagnetic complex, [Fe^II^(1-MeIm)_2_(*α*,*α*,*α*,*α*-TpivPP)], **1** (Figure 3). The first determination of the geometry of 1:1 coordinated dioxygen in an Fe(II) model compound, [Fe(O_2_)(l-MeIm)(*α*,*α*,*α*,*α*-TpivPP)], **1-O_2_**, is then crystallized by dropwise heptane addition to the benzene solution of **1** exposed to O_2_ (1 atm) in the presence of a small excess of 1-MeIm. **1-O_2_** has a similar Mössbauer spectrum to oxyhemoglobin and otherwise closely resembles biologically important heme-protein molecules. However, crystals of **1-O_2_** are too small to afford good x-ray intensity data. Hearteningly, soon afterwards, they find that toluene replaces benzene as a solvent to yield large crystals of **1-O_2_**, which allows to analyze its structure in detail [44]. Crystal structure displays that **1-O_2_** has four pivalamido groups on one side of the porphyrin forming a hydrophobic pocket of 5.4 Å depth, which encloses coordinated dioxygen in an “end-on” model. The end-on bent (Fe−O−O angle ~126°) dioxygen ligand is in close agreement with Pauling’s 1964 model for describing oxy-Hb electronic structure [41]. **1-O_2_** crystallizes in two space groups with four molecules in each unit cell, where the Fe−O−O plane is either parallel or perpendicular to the trans-axial imidazole plane. Thus, significant distortions result. The structure indicates O−O distances of 1.23(8) and 1.26(8) Å, which are consistent with that of coordinated superoxides [81].

Collman and co-workers replace 1-MeIm with 2-methylimidazole (2-MeIm), and a five-coordinate complex [Fe(2-Melm)(*α*,*α*,*α*,*α*-TpivPP)(C_2_H_5_OH)] (**2**) is synthesized. The six-coordinate dioxygen adduct of **2**, [Fe(O_2_)(2-MeIm)(*α*,*α*,*α*,*α*-TpivPP)] (**2-O_2_**) is then prepared by exposing crystals of **2** for 3 days at 20 °C to 1–2 atm of O_2_, which is saturated with ethanol vapor [81,82,83]. The 2-MeIm ligand leads to lengthened axial bonds relative to the sterically undemanding l-MeIm ligand, and the sum of the Fe-N_im_ and Fe-O separations is 4.005 Å in **2-O_2_**, but only 3.813 Å in **1-O_2_**. For **2-O_2_**, the compromise between minimum destabilizing nonbonding contacts and maximum bonding leads to the iron atom remaining 0.086 Å out of the plane toward the imidazole ligand, in contrast to **1-O_2_**, where the iron atom is displaced only 0.030 Å toward the O_2_ ligand [44,76]. The porphyrinato-dioxygen nonbonding contacts are not significantly different between **2-O_2_** and **1-O_2_**. Further adjustment for the steric hindrance of the 2-MeIm group is made by significant buckling of the porphyrinato skeleton, that is, the mean displacement from the least-squares plane is 0.066 Å in **2-O_2_**, which is 0.010 Å larger than that in **1-O_2_**. The O_2_ ligand is again found coordinated in the bent, end-on fashion, with four-fold disorder.

Suppression of irreversible oxidation was so successful with [Fe^II^(*α*,*α*,*α*,*α*-TpivPP)] when l-MeIm and 2-MeIm as the axial ligands that [Fe^II^(*α*,*α*,*α*,*α*-TpivPP)] with other axial bases has also been examined to isolate the dioxygen adducts by Collman and co-workers (Figure 3). Benzene solutions of [Fe^II^(*α*,*α*,*α*,*α*-TpivPP)] prepared under nitrogen and containing an excess of 1-*n*-BuIm are treated with O_2_ (1 atm) at 25 °C, which results in the visible spectral changes to form dioxygen adduct, [Fe(O_2_)(1*-n*-BuIm)(*α*,*α*,*α*,*α*-TpivPP)] (**3-O_2_**). The Mössbauer and magnetic data show that **3-O_2_** is essentially diamagnetic, like oxy-Hb [42,84]. **3-O_2_** does not show IR or Raman absorptions at room temperature, but at −175 °C, its IR spectrum reveals a remarkably sharp band at 1385 cm^–1^, which was assigned as dioxygen vibration (*v*_O2_). Reduction of [Fe^III^Br(*α*,*α*,*α*,*α*-TpivPP)] in THF produces crystals [85] of high-spin [Fe^II^(THF)(*α*,*α*,*α*,*α*-TpivPP)] (**4**) at 25 °C where only one THF is presumed to coordinate to iron(II). This assumption is consistent with the behavior of the simple ferrous complexes in THF solution [86]. In the solid state, **4** reversibly absorbs 1 equiv. of O_2_ to yield a reported paramagnetic (*μ*_eff_ = 2.4 BM) dioxygen adduct, [Fe(O_2_)(THF)(*α*,*α*,*α*,*α*-TpivPP)] (**4-O_2_**). Coordinated O_2_ can be removed from solid **4-O_2_** in vacuo, and the reversible solid-state oxygenation is followed through several cycles by monitoring changes in magnetic susceptibility. However, the complex is later shown by Mössbauer spectroscopy to be diamagnetic [84,85]. In addition, Collman and co-workers also observe the formation of dioxygen adduct (**5-O_2_**) upon exposure [Fe^II^(THT)(*α*,*α*,*α*,*α*-TpivPP)] (**5**; THT: tetrahydrothiophene) to O_2_. The Mössbauer spectrum of **5-O_2_** was found to be quite similar to that of **1-O_2_**. 

### 3.2. Secondary Coordination Sphere Interactions

**Figure 4:**
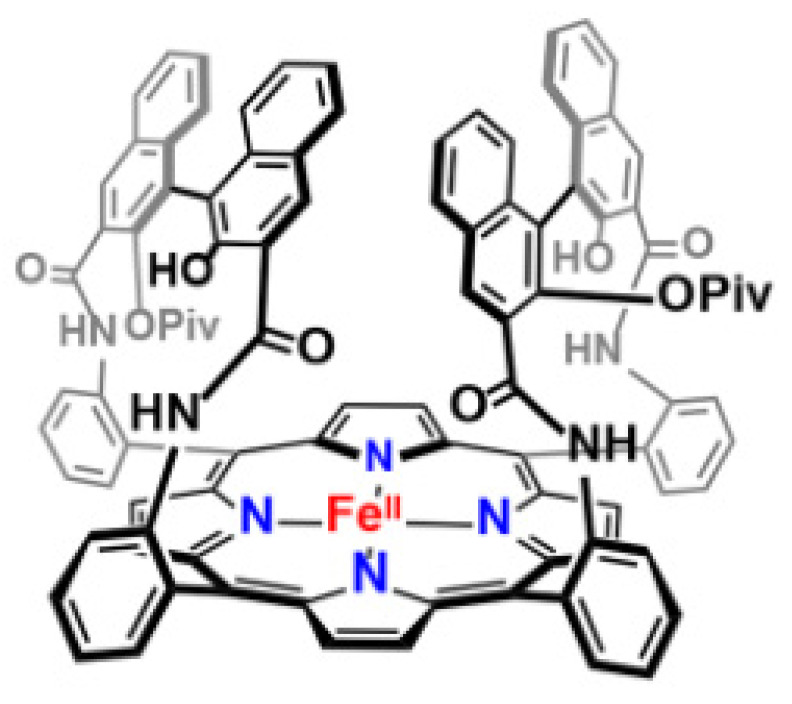
“Single-coronet” model system designed by Naruta and co-workers. Reprinted with permission from Ref. [15]. Copyright 2018, American Chemical Society.

**Figure 5:**
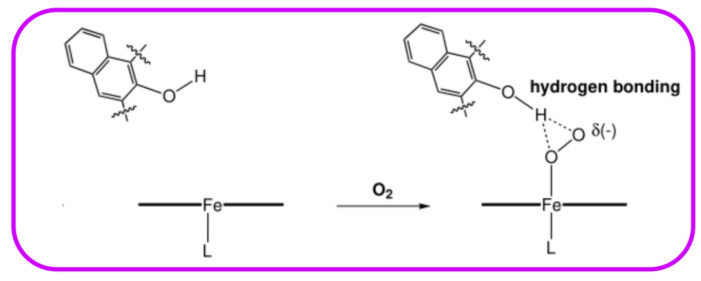
Stabilization of Fe-O_2_ species through hydrogen-bonding interactions resulting from hydroxylated naphthyl functionalities in “coronet” model systems. Reprinted with permission from Ref. [90]. Copyright 2003, WILEY-VCH Verlag GmbH.

The secondary coordination sphere interactions are another promising strategy for building discrete molecules with improved O_2_ activation properties and increasing the stability of the O_2_ intermediates. “Single-coronet” model system (**6**) designed by Naruta and co-workers is such a compound, which is constitutive of highly sterically bulky substituted dinaphthalene moieties that establish a hydrophobic environment around the Fe center, while providing –OH for secondary coordination sphere interactions (Figure 4). As presented in biological heme centers [87,88], this uniquely designed architecture has been reported to mediate the Fe-O_2_ affinities to increase the stabilities of Fe-O_2_ adducts [89,90], wherein hydrogen-bonding interactions on Fe-O_2_ adducts result in the huge change in affinity (Figure 5) [89,91].

**Figure 6:**
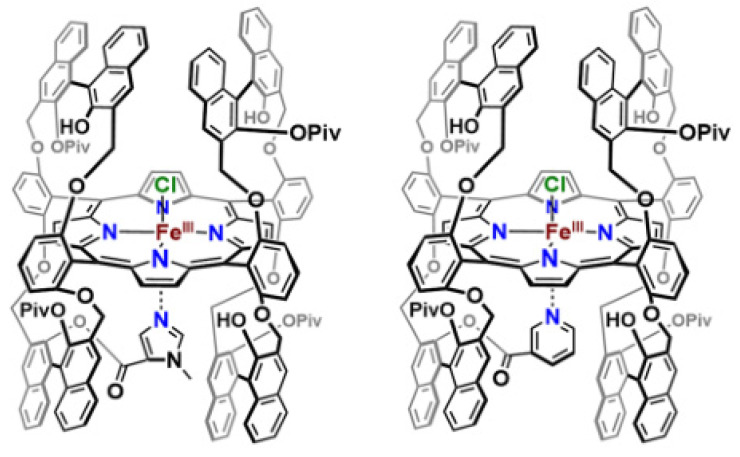
“Twin-coronet” model systems with tethered axial N-based ligands. Reprinted with permission from Ref. [15]. Copyright 2018, American Chemical Society.

In a subsequent study, Naruta and co-workers also prepare “twin-coronet” ligand platforms, which introduce a tethered imidazole (**7**) or pyridine (**8**) to the porphyrinate periphery (Figure 6) [90]. The dioxygen adducts (**7-O_2_**, **8-O_2_**) of these “twin-coronet” models reveal striking stability with half-lives up to several days in toluene at 25 °C. They suggested that the remarkable stability of the Fe-O_2_ adducts resulted from the hydrogen bonding to bound O_2_ moieties in the secondary coordination sphere in **7-O_2_** and **8-O_2_**. The resonance Raman (rR) spectra of **7-O_2_** and **8-O_2_** showed *ν*(Fe−O) stretching centered at 586 cm^−1^ and 583 cm^−1^, respectively, which are higher than those of other Fe-oxy species in model systems [92,93,94] and biological examples [95,96], resulting from hydrogen-bonding interactions between the O_2_ moiety and the –OH of the coronet ligand. The hydrogen-bonding interactions are further proved by infrared (IR) spectroscopic features of **7-O_2_** and **8-O_2_**, where the O−H IR stretching frequency of the ligand significantly loses intensity compared to **7** and **8**. Thus, the “twin-coronet” model systems are able to mediate O_2_ affinity and shed light on the importance of secondary coordination sphere interactions, which could explain how O_2_ activation occurs in enzyme systems.

### 3.3. Cryogenic Temperature

Designing model systems that can precisely mimic the electronic and geometric characteristics of iron–dioxygen adducts that form within biological heme-containing proteins remains a huge challenge for biomimetic chemists [58,72]. Heme iron(III)-superoxo intermediates can be readily produced when iron(II) porphyrin complexes are exposed to O_2_. Nevertheless, formed iron(III)-superoxo intermediates are too unstable to be isolated or observed, resulting in only a handful of oxyheme models found to date [97,98]. The cryogenic temperature technique is a very effective way to observe and isolate iron–dioxygen adducts, because the decomposition of iron–dioxygen adducts is retarded and the bimolecular reaction to form μ-peroxo derivatives is remarkably slowed at low temperature [60,73].

**Figure 7:**
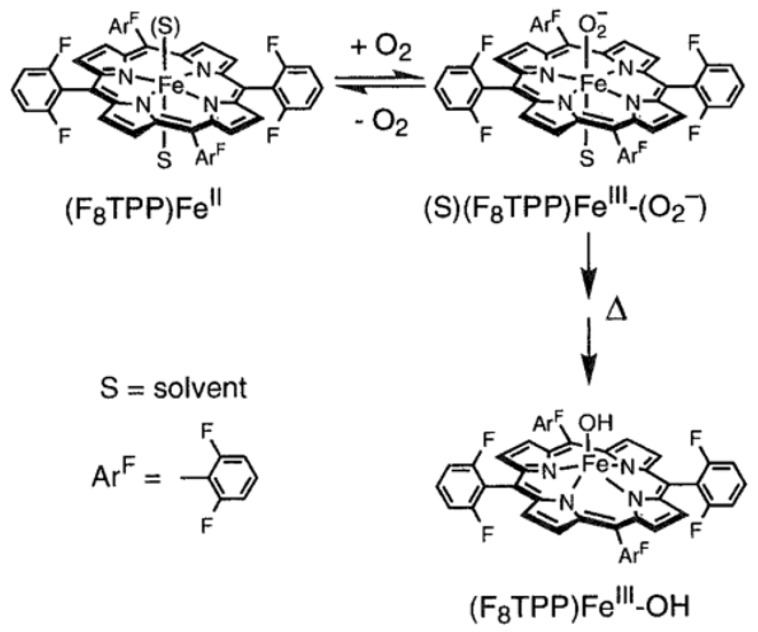
Formation of [(THF)(F_8_TPP)Fe^III^(O_2_^•−^)] and its thermal decay to [(F_8_TPP)Fe^III^(OH)]. Reprinted with permission from Ref. [100]. Copyright 2001, American Chemical Society.

Karlin and co-workers report several examples of heme−superoxo complexes, which are characterized at cryogenic temperature [99,100,101,102]. As early as in 1999, when Karlin and co-workers carry out the synthesis of an iron-copper dinuclear active site, [(F_8_TPP)Fe^III^-O-Cu^II^(MePY2)]^+^ {F_8_TPP = tetrakis(2,6-difluorophenyl)porphyrinate; MePY2 = *N*,*N*-bis [2-(2-pyridyl)ethyl]-methylamine}, they observe a transient iron(III)-superoxo species, [(F_8_TPP)Fe^III^(O_2_^•−^)] (**9-O_2_**), at cryogenic temperature by stopped-flow experiments (Figure 5). Adding O_2_ to the acetone solution including [(MePY2)Cu^I^(MeCN)]^+^ and [(F_8_TPP)Fe^II^] (**9**) at –90 °C leads to the generation of **9-O_2_** with characteristic absorption band at *λ*_max_ = 535 nm within 1 ms, which is also observed when **9** alone reacts with O_2_ in THF. However, the low-temperature stopped-flow kinetic experiments exhibit that **9-O_2_** is very unstable and transforms into a new species with distinct absorption bands at 560 nm within 1 s. Soon afterward, Karlin and co-workers reported the detailed characterization of **9-O_2_** under various conditions of solvent and temperature using multinuclear NMR and UV-visible spectroscopies in 2001 [100]. In coordinating solvents, including THF, propionitrile, and acetone, **9** reacts with O_2_ at cryogenic temperature to generate a quite stable iron(III)-superoxo species, [(S)(F_8_TPP)Fe^III^(O_2_^•−^)] (**9-S-O_2_**) (S = solvent) (Figure 7). A full reversible phenomenon is observed by benchtop UV-visible spectroscopy, the kinetics and thermodynamics of formation of **9-S-O_2_** is determined by stopped-flow spectrophotometry, and Fe/O_2_ stoichiometry of 1:1 is determined by dioxygen-uptake manometry at 193 K. The ^2^H NMR spectrum of **9-THF-O_2_** with deuterated *β*-pyrrolic positions gives evidence of a diamagnetic low-spin iron center with *δ*_pyrrole_ = 8.9 ppm, confirming the presence of a 6C Fe^III^ center in the superoxo complex, wherein the diamagnetism results from the antiferromagnetic coupling of the low-spin iron(III) ion with the superoxo radical anion [49,73]. The ^19^F-NMR spectrum of **9-THF-O_2_** reveals two identical integrations of F resonances at −111 and −113 ppm, respectively, showing two chemically different environments for the 2,6-difluoro substituents of the porphyrinate, presumably giving the credit to the two chemically inequivalent sides of the porphyrinate, wherein one bears the superoxo radical anion [68]. However, due to the instability of **9-THF-O_2_**, upon increasing temperature to room temperature, **9-THF-O_2_** decomposes to a high-spin iron(III)-hydroxide species, [(F_8_TPP)Fe^III^(OH)] (Figure 7), with a paramagnetic ^2^H NMR shift at *δ*_pyrrole_ = 125 ppm.

**Figure 8:**
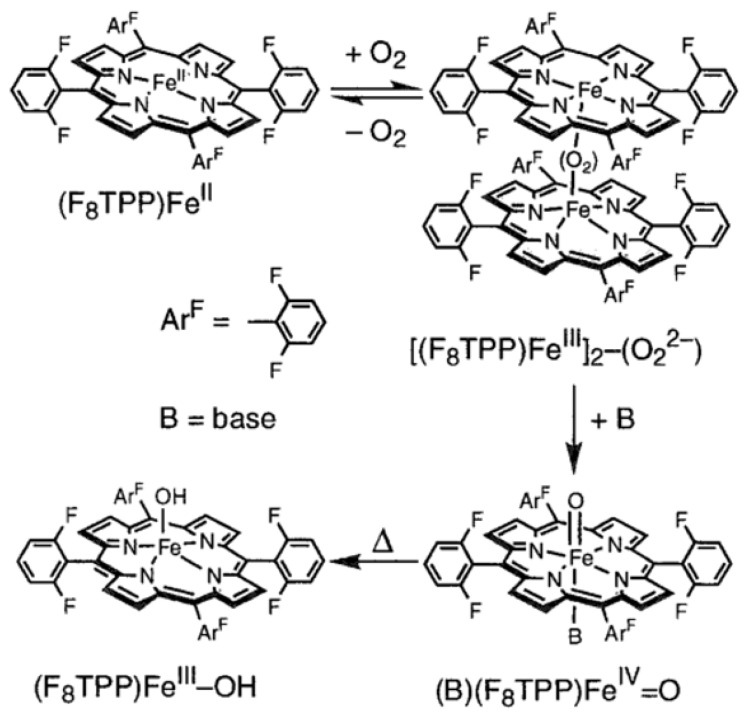
Formation of peroxo-bridged dinuclear complex {[(F_8_TPP)Fe^III^]_2_(O_2_^2−^)} in noncoordinating solvents and its stepwise transformation to a high-spin iron(III)-hydroxide species, [(F_8_TPP)Fe^III^(OH)]. Reprinted with permission from Ref. [100]. Copyright 2001, American Chemical Society.

While in noncoordinating solvents, including toluene and CH_2_Cl_2_, **9** reacts with O_2_ to generate not an iron(III)-superoxo species, but a peroxo-bridged dinuclear complex {[(F_8_TPP)Fe^III^]_2_(O_2_^2−^)} at 193 K (Figure 8) [100]. The formulation of {[(F_8_TPP)Fe^III^]_2_(O_2_^2−^)} is further corroborated by O_2_ titration of **9** in CH_2_Cl_2_ at 193 K, and a stoichiometry of 0.5−0.55 equiv of O_2_ per equiv of [(F_8_TPP)Fe^II^] is determined. {[(F_8_TPP)Fe^III^]_2_(O_2_^2−^)} transforms into a high-spin iron(III)-hydroxide species, [(F_8_TPP)Fe^III^(OH)] when increasing the reaction temperature.

### 3.4. Encapsulation of the Metalloporphyrin in Metal–Organic Frameworks (MOFs)

The aforementioned strategies to promote O_2_ activation and stabilize iron-O_2_ adducts involve complicated multistep organic synthesis and harsh reaction conditions, which limits the development of O_2_ activation. One promising approach encapsulating the metalloporphyrin in MOFs has been used as a platform to study iron–dioxygen species. MOFs are particularly well-suited for O_2_ activation because of their high degree of synthetic tunability, their porous structure, and their amenability to single-crystal diffraction studies [103,104].

**Figure 9:**
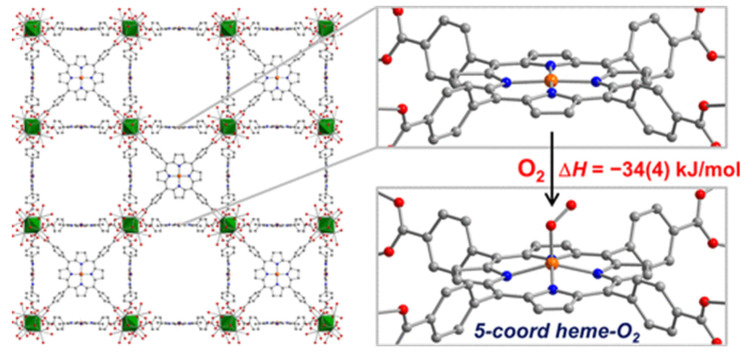
Reaction of porphyrinic MOF, PCN-224Fe^II^ (**10**), with O_2_ at −78 °C to generate the first structurally characterized 5C low-spin heme-superoxo species PCN-224Fe^III^O_2_^•−^ (**10-O_2_**). Reprinted with permission from Ref. [105]. Copyright 2014, American Chemical Society.

Recently, Harris and co-workers reported a five-coordinate heme dioxygen adduct isolated within an MOF (Figure 9) [105]. The Zr-based porphyrinic MOF PCN-224 is metalated with iron(II) to produce a four-coordinate (4C) ferrous heme-containing complex, PCN-224Fe^II^ (**10**). Even though some heme-containing MOFs have been synthesized [106,107,108,109,110], **10** is the first example of an MOF with coordinatively unsaturated ferrous heme centers. Upon exposure of single crystals of **10** to 1 atm of O_2_ at −78 °C, an obvious color change was observed. Its X-ray diffraction analysis reveals that an iron(III) center coordinates to superoxo moiety (PCN-224Fe^III^O_2_^•−^, **10-O_2_**) in an end-on *η*^1^ geometry with Fe−O distance of 1.79(1), O−O distance of 1.15(4) Å, and Fe−O−O angle of 118°. Subsequent Mössbauer spectroscopy further demonstrates the presence of a low-spin electronic configuration for iron(III). Temperature-dependent experiments reveal stoichiometry 1:1 of Fe:O_2_ and also provide insight into the energetics of dioxygen binding, giving a binding enthalpy of −34(4) kJ/mol. The binding enthalpy of **10-O_2_** is notably lower than that of 6C Fe^II^ centers (−63 to −65 kJ/mol). **10-O_2_** is the only example with a structurally characterized “base-free” 5C heme-superoxo species. These results certify the solid-state structure of MOFs can provide a platform to enable isolation and thorough characterization of unstable species that can only be observed transiently in molecular form. In their subsequent works, this system is further extended to PCN-224(Co) and PCN-224(Mn), which also demonstrated selective reversible O_2_ binding to generate Co^III^-O_2_^•−^ adduct and Mn^IV^-O_2_^2–^ adduct, respectively [111,112].

## 4. Conclusions

Dioxygen activation to generate the reactive metal–oxygen species is extremely important in the context of energy storage and utilization. In nature, a variety of metalloenzymes with cheap metal active sites, such as iron, are able to activate dioxygen under ambient conditions. These systems inspire bioinorganic chemists to synthesize biomimetic small-molecule iron porphyrin complexes, which are designed to carry out the O_2_ activation. However, the isolation and characterization of iron–dioxygen adducts is a big challenge because the absence of surrounding protein superstructure makes iron–dioxygen adducts unstable. The last half century has seen the development of biomimetic chemistry to overcome this challenge. From a historical standpoint, this review shows the great progress in the development of a variety of iron porphyrin models of O_2_-dependent heme enzymes to interpret iron–dioxygen intermediates. Owing to increasingly sophisticated spectroscopic techniques (e.g., Mössbauer, XAS, EXAFS, EPR, rR), cryogenic temperature techniques, and porphyrin ligand development, a few iron–dioxygen species are spectroscopically observed by reacting iron porphyrin complexes with O_2_. Significant sterically hindered iron porphyrin complexes with axial ligands could mimic enzymes to make metal active sites sequestered, promote O_2_ binding, and increase the stability of iron–dioxygen species. Secondary coordination sphere interaction is another useful strategy to stabilize iron–dioxygen species via H-bond interactions, which helps keep iron–dioxygen species separate from each other. However, there are limits to these strategies because they involve complicated multistep organic synthesis and harsh reaction conditions. Very recently, due to the merit of MOFs—which are well-suited for O_2_ activation because of their high degree of synthetic tunability, their porous structure, and their amenability to single-crystal diffraction studies—one promising approach encapsulating and separating the iron porphyrin in MOFs has been used as a platform to study iron–dioxygen species. The synthesis of MOFs is facile, and the encapsulation results in the trapping of Fe−O_2_ adducts that lack a sixth axial ligand trans to the O_2_ binding sites.

Metalloenzymes can efficiently catalyze O_2_-dependent oxidations, but their mechanisms remain unclear. The development of synthetic models of metal–oxygen intermediates help to illustrate the enzyme mechanisms. Meanwhile, progress in the illustration of the mechanisms for the metalloenzymes guides chemists to design potent and selective catalysts. Although O_2_ activation by biomimetic heme complexes has always been a focus of biomimetic chemists, there are still relatively few synthesized complexes that are capable of activating O_2_ so far, and the oxidation yields of these few successful complexes remain poor compared with that of metalloenzymes. With the great progress being made over the past several decades, the future holds remarkable promise in isolating metal–dioxygen intermediates in O_2_ activation by metal porphyrin complexes.

## Data Availability

Not applicable.
